# Genetic diversity and multidrug resistance of phylogenic groups B2 and D in *InPEC* and *ExPEC* isolated from chickens in Central China

**DOI:** 10.1186/s12866-022-02469-2

**Published:** 2022-02-18

**Authors:** Qin Lu, Wenting Zhang, Ling Luo, Honglin Wang, Huabin Shao, Tengfei Zhang, Qingping Luo

**Affiliations:** 1 Key Laboratory of Prevention and Control Agents for Animal Bacteriosis (Ministry of Agriculture and Rural Affairs), Institute of Animal Husbandry and Veterinary, Hubei Academy of Agricultural Sciences, Wuhan, 430064 China; 2 Hubei Provincial Key Laboratory of Animal Pathogenic Microbiology, Institute of Animal Husbandry and Veterinary, Hubei Academy of Agricultural Sciences, Wuhan, 430064 China

**Keywords:** *Escherichia coli*, Phylogenic group, MLST, Antimicrobial resistance pattern, Virulence-associated genes

## Abstract

**Background:**

Avian colibacillosis is an infectious bacterial disease caused by avian pathogenic *Escherichia coli* (APEC). APEC causes a wide variety of intestinal and extraintestinal infections, including *InPEC* and *ExPEC*, which result in enormous losses in the poultry industry. In this study, we investigated the prevalence of *InPEC* an*d ExPEC* in Central China, and the isolates were characterized using molecular approaches and tested for virulence factors and antibiotic resistance.

**Results:**

A total of 200 chicken-derived *E. coli* isolates were collected for study from 2019 and 2020. The prevalence of B2 and D phylogenic groups in the 200 chicken-derived *E. coli* was verified by triplex PCR, which accounted for 50.53% (48/95) and 9.52% (10/105) in *ExPEC* and *InPEC*, respectively. Additionally, multilocus sequence typing method was used to examine the genetic diversity of these *E. coli* isolates, which showed that the dominant STs of *ExPEC* included ST117 (*n* = 10, 20.83%), ST297 (*n* = 5, 10.42%), ST93 (*n* = 4, 8.33%), ST1426 (n = 4, 8.33%) and ST10 (*n* = 3, 6.25%), while the dominant ST of *InPEC* was ST117 (*n* = 2, 20%). Furthermore, antimicrobial susceptibility tests of 16 antibiotics for those strains were conducted. The result showed that more than 60% of the *ExPEC* and *InPEC* were resistant to streptomycin and nalidixic acid. Among these streptomycin resistant isolates (*n* = 49), 99.76% harbored aminoglycoside resistance gene *strA*, and 63.27% harbored *strB*. Among these nalidixic acid resistant isolates (*n* = 38), 94.74% harbored a S83L mutation in *gyrA*, and 44.74% harbored a D87N mutation in *gyrA*. Moreover, the prevalence of multidrug-resistant (MDR) in the isolates of *ExPEC* and *InPEC* was 31.25% (15/48) and 20% (2/10), respectively. Alarmingly, 8.33% (4/48) of the *ExPEC* and 20% (2/10) of the *InPEC* were extensively drug-resistant (XDR). Finally, the presence of 13 virulence-associated genes was checked in these isolates, which over 95% of the *ExPEC* and *InPEC* strains harbored *irp2*, *feoB*, *fimH*, *ompT*, *ompA*. 10.42% of the *ExPEC* and 10% of the *InPEC* were positive for *kpsM*. Only *ExPEC* isolates carried *ibeA* gene, and the rate was 4.17%. All tested strains were negative to *LT* and *cnf* genes. The carrying rate of *iss* and *iutA* were significantly different between the *InPEC* and *ExPEC* isolates (*P* < 0.01).

**Conclusions:**

To the best of our knowledge, this is the first report on the highly pathogenic groups of *InPEC* and *ExPEC* in Central China. We find that 50.53% (48/95) of the *ExPEC* belong to the D/B2 phylogenic group. The emergence of XDR and MDR strains and potential virulence genes may indicate the complicated treatment of the infections caused by APEC. This study will improve our understanding of the prevalence and pathogenicity of APEC.

**Supplementary Information:**

The online version contains supplementary material available at 10.1186/s12866-022-02469-2.

## Background

Avian pathogenic *Escherichia coli* (APEC) is responsible for a variety of extra-intestinal pathogenic effects in poultry. The most common lesions observed on gross postmortem of avians with systemic colibacillosis include airsacculitis, pericarditis, and perihepatitis [1, 2]. Most *E. coli* are commensal bacteria colonizing in the gut. However, pathogenic *E. coli* can cause various infections in the intestinal system and the bloodstream. Based on whether the disease syndrome is intra- or extra-intestinal, pathogenic *E. coli* can be classified into *InPEC* and *ExPEC* [3]. Recent studies have reported significant differences in the evolutionary tree, drug resistance, sequence types (ST) and virulence genes of *E. coli* isolates obtained from humans and poultry in China and elsewhere, but there are very few reports regarding *InPEC* and *ExPEC* in Central China.

The phylogenetic classifications of *InPEC* and *ExPEC* were significantly different. Najafi et al. showed that commensal *E. coli* that survive within the intestinal system mainly belong to the A/B1 group, while those in the highly pathogenic *ExPEC* are generally in the B2/D group [4]. A similar study with 994 avian isolates conducted by Johnson et al. showed that all of which were highly pathogenic and capable of causing colisepticemia [5]. Sen et al. [6] analyzed samples from crow feces and water in their wetland habitat. They showed that crows were the carriers of *ExPEC* and APEC-like strains and the majority of *ExPEC* isolates were associated with *E. coli* phylogenetic groups B2 and D. Pathogenic *E. coli* found in humans and poultry carcasses showed similar virulence and resistance [7].

A variety of APEC virulence factors determine their pathogenicity. The virulence factor genes *iutA* and *iroN* (iron metabolism), *iss* (increased serum survival), *hlyF* (hemolysis), and *ompT* (surface exclusion and serum survival) could be present on large plasmids, a defining and necessary trait for APEC virulence [3]. In addition to these plasmid associated genes, APEC isolates were also characterized by the possession of certain chromosomally encoded virulence genes including *fyuA* (yersiniabactin receptor), pap operon genes (*papA*, *papC*, *papEF*, and *papG* that encode parts of the P pilus), and *ibeA* (pathogenicity island markers) [8, 9]. Stromberg et al. [10] showed that the distribution of *papA*, *papC*, *papEF*, *papG2*, *papG3*, *kpsM II*, and *tsh* was significantly different (*P* < 0.001) between *ExPEC* (*n* = 40) and non-*ExPEC* (*n* = 37) samples. Additionally, they found that some *E. coli* isolates from feces of healthy chicken had *ExPEC* virulence-associated genes, which could cause *ExPEC*-associated illness in animal models. Their study showed that the *E. coli* isolates containing *ExPEC*-associated genes might contribute to the chicken-to-chicken *ExPEC* transmission through pecking or inhalation of contaminated fecal dust. These isolates may ultimately result in severe poultry disease or death. Not only causing disease in chicken, but a recent study also showed that the prevalence of *ibeA* indicated a closer relationship between APEC and newborn meningitic (NMEC) strains, which has a zoonotic potential and presents a significant health risk to humans [11].

MLST (Multilocus sequence typing) data from previous studies have shown that ST10, ST48, ST95, and ST117 predominate among the *E. coli* STs found in poultry [12]. The sequence types of the *ExPEC* isolates belonging to the B2 phylogenetic group were analyzed by MLST, showing the most prevalent genotypes were ST131, ST95, ST14, ST10, ST69, ST1722, ST141, ST88, ST80, and ST99 8[13]. Genotypic analysis of *E. coli* isolates from pigs with diarrhea in China revealed that the most prevalent genotypes were ST10 and ST48, followed by ST29, ST744, ST101, ST4214, and ST61 7[14]. The most prominent genotype was ST117, ST2847 and ST48 in APEC [15].

Drug-resistant APEC strains can contaminate the food supply from farm to fork through eggs, meat, and other commodities and thus pose a severe threat to consumer health [16]. Inappropriate selection and abuse of antibiotics in the poultry industry may have contributed to drug resistance in APEC. The *E. coli* isolates considered resistant using the cut-offs provided by the Clinical and Laboratory Standards Institute (CLSI), while the intrinsic resistance needs to be addressed by multidrug-resistant (MDR), extensively drug-resistant (XDR), pan drug-resistant PDR [17]. Hirakata showed China faced the highest rate of antimicrobial resistant (AMR) among all Asian countriers [18]. China utilizes the largest quantity of antibiotics worldwide. Almost 30% of drugs are sold to China. This proportion of antibiotic usage is about 20% higher than the developed countries [19]. Many researchers reported the MDR of cephalosporins, quinolones, aminoglycosides, and penicillin among the *E. coli* strains isolated from animals and humans, which reflected the extensive and heavy use of these antibiotics in Western and Eastern China [20–22].

Therefore, it is important to study the relationships between the genetic evolution of *InPEC* and *ExPEC* isolates and their pathogenicity and drug resistance to improve our understanding of the prevalence and pathogenicity of APEC.

## Results

### Classifying of phylogenetic groups of *E. coli* isolates

**Fig. 1:**
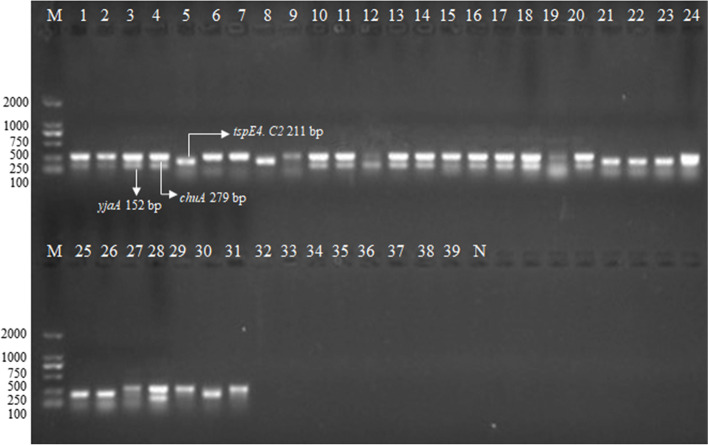
The certification of phylogenetic groups of partial *E. coli* isolates by PCR amplification. M. 2000 bp DNA ladder; B2 phylogenetic group (*chuA*^+^ *yjaA*^+^ *tspE4.C2*^−^), lane 1, 2, 3, 4, 10, 11, 13, 14, 15, 16, 17, 18, 19, 20, 28; D phylogenetic group (*chuA*^+^ *yjaA*^−^ *tspE4.C2*^−^), Lane 6, 7, 9, 27, 29, 31; B1 phylogenetic group (*tspE4.C2*^+^ *chuA*^−^ *yjaA*^−^), Lane 5, 8, 12, 21, 22, 23, 24, 25, 26, 30; A phylogenetic group (*chuA*^−^ *yjaA*^−^ *TspE4.C2*^−^), Lane 32, 33, 34, 35, 36, 37, 38, 39; N. negative control, Lane 40

A phylogenetic group analysis of 200 *E. coli* from chicken revealed that most of the *E. coli* strains isolated belonged to group A (*n* = 92, 46%), followed by groups B1 (*n* = 50, 25%), B2 (*n* = 18, 9%), and D (*n* = 40, 20%), which suggested that 58 isolates (B2 and D) were APEC. A total of 58 APEC were divided into 48 *ExPEC* from sick and diseased chickens, included liver (*n* = 26), brain (n = 9), heart (n = 5), lung (*n* = 3), eye (n = 1), breast (n = 1) and stomach (n = 1) in Xiannin, Jiangxia, Xiangyang, Shishou, and Yichang cities, and 10 *InPEC* from diseased broiler chickens: feces (n = 2), intestine (*n* = 8) in Xiannin, Jiangxia, Tianmen, Xinzhou, Xiangyang cities. The determination of *E. coli* phylogenetic groups showed that the majority of the 95 isolates from extra-intestinal tissues belonged to phylogenetic group D (*n* = 34, 35.8%), followed by groups A (*n* = 26, 27.4%), B1 (*n* = 21, 22.1%), and B2 (*n* = 14, 14.7%), while the majority of the 105 isolates from feces and intestines belonged to phylogenetic group A (*n* = 66, 62.9%), followed by groups B1 (*n* = 29, 27.6%), D (n = 6, 5.7%), and B2 (*n* = 4, 3.8%) (Fig. 1) (Table 1). These results showed that the *E. coli* from feces and intestines mostly belong to group A, while the *E. coli* from extra-intestinal tissues mostly belong to group D. The distribution of occurrence of groups A, B2, and D were significantly different between *E. coli* isolates from extra-intestinal (27.4, 14.7, and 35.8%, respectively) and intestinal tissues (62.9, 3.8, and 5.7%, respectively) in our study (*P* < 0.01).

**Table 1:** Phylogenetic distribution of extra-intestinal and intestinal *E. coli* isolates

Phylogenetic type	Extra-intestinal *E. coli* (*n* = 95)	Intestinal *E. coli* (*n* = 105)	*P* value	Total (%)
A	26 (27.4%)	66 (62.9%)	0	92 (46%)
B1	21 (22.1%)	29 (27.6%)	0.513	50 (25%)
B2	14 (14.7%)	4 (3.8%)	0.002	18 (9%)
D	34 (35.8%)	6 (5.7%)	0	40 (20%)

### MLST of genetic diversity of *InPEC* and *ExPEC*

**Fig. 2:**
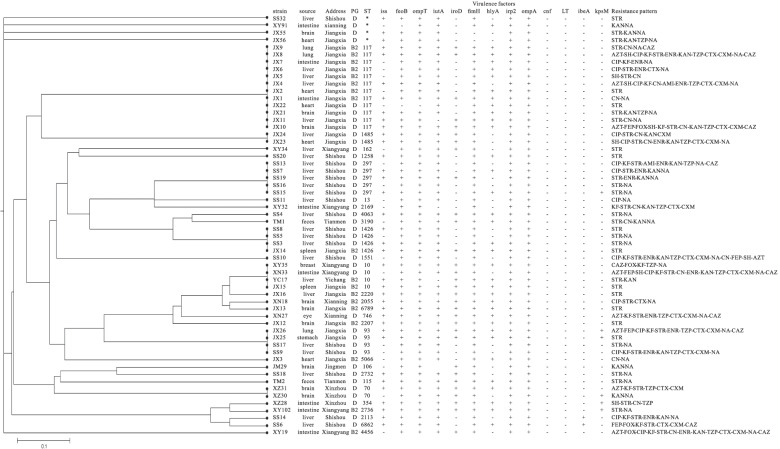
MLST minimum evolution tree, virulence gene, resistance patterns of the high-phylogenetic groups of *E. coli* isolates

The genetic diversity of all isolates belonged to high phylogenetic groups (58 isolates, groups B2 and D), including 48 *ExPEC* and 10 *InPEC*, was analyzed by MLST. The results showed that 58 strains of *InPEC* and *ExPEC* contained 29 STs (Fig. 2). The dominant phylogenetic genotype group in *InPEC* isolates was ST117 (20%), and the percent of other STs was all 10%, included ST4456, ST354, ST2736, ST115, ST10, ST2169, and ST3190. The *ExPEC* isolates were divided into 22 STs. The dominant genotypes were ST117 (20.83%), ST297 (10.42%), ST93 (8.33%), ST1426 (8.33%), ST10 (6.25%), ST1485 (4.17%), and ST70 (4.17%), while the single occurrence of 15 other STs was identified, including ST162, ST1258, ST13, ST4063, ST1551, ST2220, ST2055, ST6789, ST746, ST2207, ST5066, ST106, ST2732, ST2113, and ST6862, indicating the high genetic diversity of *ExPEC* (Fig. 2). ST117 had the widest distribution in *InPEC* and *ExPEC* isolates.

### Distribution of virulence genes in *InPEC* and *ExPEC*

The distribution of 13 virulence genes in 58 strains of *InPEC* and *ExPEC* has been examined in this study. As shown in Table 2, *cnf and LT* were not found in all isolates, while the other 11 virulence-associated genes, including *irp2*, *ompA*, *feoB*, *ompT*, and *fimH*, were found in most of the *InPEC* and *ExPEC* isolates. Additionally, the presence of *kpsM* was low in *InPEC* (10%) and *ExPEC* (10.42%). *ibeA* was not detected in *InPEC* isolates and was found in only two isolates of *ExPEC*. The presence of *iroD* and *hlyA* was 50–70% in both *InPEC* and *ExPEC* isolates, while the distribution of *iss* and *iutA* was significantly difference between *InPEC* and *ExPEC* isolates (*P* < 0.01).

**Table 2:** Prevalence of virulence-associated genes between *InPEC* and *ExPEC isolates*

Functional category	Gene	No. of isolates positive(%)		*P*-value ^a^
		*ExPEC*(*n* = 48)	*InPEC*(n = 10)	
Iron chelation	*feoB*	47 (97.92)	9 (90.00)	0.318
	*iutA*	46 (95.83)	6 (60.00)	0.006
	*iroD*	24 (50.00)	7 (70.00)	0.311
	*irp2*	48 (100.00)	10 (100.00)	1.000
Adhesin	*fimH*	46 (95.83)	10 (100.00)	1.000
Protectins	*ompT*	47 (97.92)	10 (100.00)	1.000
	*ompA*	48 (100.00)	10 (100.00)	1.000
	*hlyA*	29 (60.42)	5 (50.00)	0.726
Toxin	*cnf*	0 (0.00)	0 (0.00)	NT
	*LT*	0 (0.00)	0 (0.00)	NT
Miscellaneous	*ibeA*	2 (4.17)	0 (0.00)	1.000
Protectins	*iss*	34 (70.83)	2 (20.00)	0.004
	*kpsM*	5 (10.42)	1 (10.00)	1.000

^a^ *P*-values determined by Fisher’s exact test, two-tailed

### Antimicrobial resistant patterns in *InPEC* and *ExPEC*

The differences in the distribution of antimicrobial resistant patterns between 58 strains of *InPEC* and *ExPEC* in our study were tested against 16 types of antimicrobial agents. Most of the *InPEC* and *ExPEC* isolates were resistant to nalidixic acid (87.5% in *ExPEC* and 60% in *InPEC*), streptomycin (87.5% in *ExPEC* and 70% in *InPEC*), gentamicin (18.75% in *ExPEC* and 60% in *InPEC*), and kanamycin (33.33% in *ExPEC* and 50% in *InPEC*). A few of the *InPEC* and *ExPEC s*howing the lowest resistance rate, which were cefoxitin (2.1% in *ExPEC* and 30% in *InPEC*), cefepinme (8.33% in *ExPEC* and 10% in *InPEC*), and amikacin (4.17% in *ExPEC* and 0% in *InPEC*) (Table 3). Notably, 29.31% (17/58) of the isolates were resistant to at least 3 different types of antibiotics and classified as multidrug-resistant (MDR) strains. The 17 isolates presented 11 different types of antibiotic resistance patterns. Additionally, 10.34% (6/58) of the isolates remain susceptible to only one or two antimicrobial agents, categorized as XDR, including two types of antibiotic resistance patterns (Table 4).

**Table 3:** Distribution of resistance phenotypes and antimicrobial resistance genes detected in *InPEC* and *ExPEC* isolates

Antimicrobial classes	Antimicrobial agents	APEC(*n* = 58)		Resistance genes
		*InPEC*	*InPEC*	(*n* = 58)
		(*n* = 48)	(*n* = 10)	
β-lactamases	Cefepime	4 (8.33)	1 (10.00)	*TEM*(1, 1.72%) *SHV*(24, 41.38%) *OXA*(3, 5.17%) *CTX-M*(33,56.9%)
	Cefotaxime	12 (25.00)	3 (30.00)	
	Ceftazidime	8 (16.67)	2 (20.00)	
	cephalothin	12 (25.00)	4 (40.00)	
	cefuroxime	11 (22.92)	3 (30.00)	
	piperacillin	13 (27.08)	4 (40.00)	
	Cefoxitin	3 (6.25)	1 (10.00)	
	Aztreonam	7 (14.58)	2 (20.00)	
Aminoglycosides	Amikacin	2 (4.17)	0 (0.00)	*strA*(48, 82.76%) *strB*(31, 53.45%) *aadA* (1, 1.72%)
	Kanamycin	16 (33.33)	5 (50.00)	
	Streptomycin	42 (87.50)	7 (70.00)	
	Gentamicin	9 (18.75)	6 (60.00)	
	Spectinomycin	6 (12.50)	2 (20.00)	
Fluoroquinolones/Quinolones	Ciprofloxacin	13 (27.08)	3 (30.00)	*gyrA*(S83L) (36, 62.06%) *gyrA*(D87N) (17, 29.31%)
	Enrofloxacin	12 (25.00)	3 (30.00)	
	Nalidixic acid	30 (62.50)	8 (80.00)

**Table 4:** The MDR/ XDR phenotype of *InPEC* and *ExPEC* isolates against antimicrobial agents of different classes

Type of resistance	Resistance patterns	Numer		
		***ExPEC***	***InPEC***	total
**XDR**	**Resistance to at least one antimicrobial agents of eight antimicrobial classes** CTX/ CTX, CAZ + KF/ KF, CXM + TZP + FOX + AZT + STR, CN, KAN, SH + CIP, ENR + NA	0	1	1
**XDR**	**Resistance to at least one antimicrobial agents of seven antimicrobial classes** CTX/ FEP, CTX, CAZ/ CTX, CAZ + KF, CXM + TZP + AZT + STR/ STR, CN, KAN, SH/ STR, SH, KAN/ SH, CN, AMI + CIP, ENR/ ENR + NA	4	1	5
**MDR**	**Resistance to at least one antimicrobial agents of six antimicrobial classes** CAZ/ CTX + KF/ KF, CXM + TZP + STR, KAN/ STR, KAN, AMI / STR, SH, CN, KAN + ENR / ENR, CIP + NA FEP, CTX, CAZ + KF, CXM + TZP + FOX + AZT + STR, SH, CN, KAN	3	0	3
**MDR**	Resistance to at least one antimicrobial agents of five antimicrobial classes CAZ + KF + TZP + FOX +NA CTX + KF, CXM + TZP + AZT + STR	2	0	2
**MDR**	**Resistance to at least one antimicrobial agents of four antimicrobial classes** CTX + STR + CIP / CIP, ENR + NA FEP, CTX, CAZ + KF, CXM + FOX + STR CTX + KF, CXM + TZP + ARE, CN, KAN KF + STR, KAN + ENR, CIP + NA	4	1	5
**MDR**	Resistance to at least one antimicrobial agents of three antimicrobial classes TZP + STR, KAN + NA CAZ + STR, CN + NA KF / STR / STR, KAN / CN + CIP, ENR + NA	6	1	7

Resistance breakpoints were Ceftazidime (CAZ, ≤17 mm), Cefoxitin (FOX, ≤14 mm), Cefepime (FEP, ≤14 mm), Cefotaxime (CEF,≤22 mm), Cefuroxime (CXM, ≤14 mm), Piperacillin (TZP, ≤17 mm), Aztreonam (AZT, ≤17 mm), Cephalothin (KF, ≤14 mm), Amikacin (AMI, ≤12 mm), Kanamycin (KAN, ≤13 mm), Streptomycin (STR, ≤11 mm), Gentamicin (CN, ≤12 mm), Spectinomycin (SH, ≤14 mm), Ciprofloxacin (CIP, ≤15 mm), Nalidixic acid (NA, ≤13 mm), Enrofloxacin (ENR, ≤15 mm) (Clinical and Laboratory Standards Institute, 2017)

#### Detection of antimicrobial resistance genes

Among the 58 strains of *InPEC* and *ExPEC*, aminoglycoside resistant genes *strA*, *strB*, and *aadA* were found in 82.76, 53.45, and 1.72% of these isolates respectively, and all of the streptomycin resistant isolates (*n* = 49) contained one or more of these resistance genes. Of the β-lactamases resistant genes, *CTX-M* was the most prevalent gene (56.90%), followed by *SHV* (41.38%), *OXA* (5.17%) and *TEM* (1.72%). The S83L mutation and D87N mutation in *gyrA*, which could cause quinolones/fluoroquinolones resistance, were found in 62.06 and 29.31% of our *InPEC* and *ExPEC* isolates. Among these streptomycin nalidixic acid resistant isolates(*n* = 38), 94.74% contained S83L mutation in *gyrA* (Table 3, Supplement Table 1).

## Discussion

Poultry and their products are commonly consumed by humans, but little detailed information is available regarding the *InPECs* and *ExPECs* isolated from poultry in Central China. In our study, we found that 29% of the 200 clinical samples belonged to high phylogenetic groups of *InPEC* and *ExPECs*, posing a severe threat to consumer health. *ExPECs* cause multi-system mixed infections in avians, including myocarditis, septicemia, perihepatic and balloon inflammations. Most of the *ExPECs* belonged to groups B2 and D, especially the highly pathogenic strains in group B2, while the symbiotic *E. coli* and *InPEC* were mainly in groups A and B1. Although many investigators have shown that APEC is a type of *ExPEC*, this study is the first to distinguish the relationship between *ExPEC* and *InPEC* in groups B2 and D of APEC. The distribution of phylogenetic groups showed that the majority of the 95 extra-intestinal *E. coli* belonged to phylogenetic group D (35.8%), followed by groups A (27.4%), B1 (22.1%), and B2 (14.7%), and the majority of the 105 intestinal *E. coli* belonged to phylogenetic group A (62.9%), followed by groups B1 (27.6%), D (5.7%), and B2 (3.8%), the results that are consistent with Amani F [17]. Hussain et al. [23] and Huja et al. [18] analyzed the entire genome sequences of 28 *E. coli* isolates from 12 broiler chickens (ten from the cecum and two from meat), 11 free-range chickens (six from the cecum and five from meat), and five human *ExPEC* isolates. The results showed that *E. coli* from the free-range chicken belonged to group B1, and the human *ExPEC* isolates and two isolates of *E. coli* from broiler meat belonged to groups B2 and D, suggesting that phylogroups B2 and D represent significant potential zoonotic factors in *ExPEC*.

Potential virulence properties include adhesion and invasion of epithelial cells, secretion of iron, serum resistance, and the formation of toxins. To better understand the virulence potential of *E. coli* isolates, we characterized 13 virulence-associated genes in five types of virulence functions. Iron chelation, which captures trivalent iron (Fe^3+^) from ferritin and transferrin, plays an essential role in *ExPEC* virulence. Evaluation of *E. coli* isolates from healthy chickens, to determine their potential risk to poultry and human health, found the risk genes *irp2*, *feoB*, and *iutA* in more than 89% of the strains isolated, and significant differences in the occurrence of *iutA* between *InPEC* and *ExPEC* (*P* < 0.01). APEC strains could transmit from avian to humans by improperly prepared poultry meat and direct contact with avians and their feces. In addition, the APEC strains may constitute a reservoir of antibiotic-resistant bacteria and pose a potential risk to human health [24, 25]. Amani et al. examined the distribution of *irp2* in 45 *InPEC* and 84 *ExPEC* isolates from ostriches [26]. They found that 4.4% of the *ExPEC* isolates contained the *irp2* gene compared with 27.9% in *InPEC* (*P* < 0.01), while 22.2% of *InPEC* isolates had the *iss* gene compared with 25% in *ExPEC* isolates. Huja et al. showed that the *iss* gene was conserved in all the *E. coli* septicemic strains and demanded for septicemia [27]. They also reported that *feoB* existed in all 48 *E. coli* isolates, and that 59.3% of *ExPEC* contained the *irp2* gene compared with 27.9% in *InPEC* isolates (*P* < 0.01). In the Sistan region of Iran, marker genes of *iss* and *irp2* genes were used to differentiate *InPEC* and *ExPEC* strains to improve colibacillosis control measurements [28]. In our study, over 90% of *InPEC* and *ExPEC* strains contained *irp2* and *feoB,* and 70.8% of *ExPEC* isolates contained the *iss* gene compared with 20% of *InPEC* isolates (P < 0.01). The deletion of *kpsM* in *E. coli* decreased its virulence in pigs and reduced its adhesion, phagocytosis, and serum bactericidal survival [29]. The loss of *kpsM* decreased the pathology scores in the ileum and ceca of mice [30]. In our study, 4.2% of *ExPEC* strains contained *ibeA* compared with 0% in *InPEC,* and 10.4% of *ExPEC* had the *kpsM* gene compared with 10% in *InPEC*, which are in partial agreement with the studies mentioned above.

Phylogenic groups of B2 and D from *ExPEC* is relation to ST lineages are common worldwide. The most prevalent lineages of *ExPEC* in the UK are ST131/B2, ST127/B2, ST95/B2, ST73/B2, and ST69/D. The most widespread lineages of *InPEC* in Hunan Province were ST95 and ST131 [31]. ST117 can mediate the expression of the resistance genes, such as *vanB*, *foasA*, and *CTX-M*, and is related to serotypes such as O_111_ and O_78_ in *Enterococcus faecium* [32–35]. Mora et al. studied a human septicemic O_111_:H_4_-D-ST117 *ExPEC* strain in 2000 and 200 9[36]. Their study demonstrated the slow evolution based on virulence-gene differences and macrorestriction profiles and suggested ST117 as a candidate for the development of a future vaccine against avian colibacillosis. In our study, the majority of MLSTs of *ExPEC* were ST117, ST10 and ST70, and the major MLSTs of *InPEC* were ST117, ST4456, ST354, ST2736, ST115, ST10, ST2169, and ST3190. To the best of our knowledge, this is the first report of the phylogenic groups of B2 and D of *InPEC* and *ExPEC* in Central China. Fourteen different sequence types were identified, with ST117 (16%), ST2847 (10.7%), and ST48 (5.3%) being the most prevalent. ST117 can cause colibacillosis and is a highly pathogenic group whose horizontal transmission poses potential threats to human and bird health.

Antibiotic-resistant strains of *E. coli* have been reported to cause more severe disease in humans, and the emergency of multidrug-resistant strains increases the threat to public safety. Fluoroquinolones, aminoglycosides and β-lactams are frequently used as therapeutic drugs in the treatment to severe cases. High fluoroquinolone and tetracycline-resistance rates have also been reported in other studies in China and other countries. The genomic landscape of 75 APEC isolates in Pakistan predicted that the percentage of the resistance genes against aminoglycosides, tetracyclines, sulfonamides, and beta-lactams was 89.33, 89.3, 89.3 and 88%, respectively [15]. Genomic analysis of APEC isolates from Central European countries revealed the predominant multi-antibiotic resistance genes conferring resistance against beta-lactams (28.1%), tetracyclines (37.5%) and sulfonamides (25%). Seventy-nine APEC isolates showed high resistance to ampicillin (83.5%), nalidixic acid (65.8%), tetracycline (64.6%), cephalothin (46.8%), and ciprofloxacin (46.8%) [37]. All of the 116 APEC isolated in Eastern China showed high resistance to ampicillin (100%), tetracycline (100%), nalidixic acid (89.62%), chloramphenicol (83.96%), and kanamycin (80.19%). Most of the *InPEC* and *ExPEC* isolates in our study in Central China were resistant to KF (90% in *ExPEC* and 100% in *InPEC*), cefotaxime (90% in both *ExPEC* and *InPEC*), cefuroxime (81% in *ExPEC* and 100% in *InPEC*), and kanamycin (69% in *ExPEC* and 70% in *InPEC*)*. E. coli* isolated from frozen chicken meat showed resistance rates of 95.8, 90.4, and 76.7% against cefepime, cefoxitin, and cefotaxime, respectively [38], while the rates to cefotaxime in *ExPEC* and *InPEC* were 25 and 30% in our study. The resistance rate to aminoglycosides was quite low in our study (gentamicin, 18.75% in *ExPEC* and 60% in *InPEC*; spectinomycin, 12.5% in *ExPEC* and 20% in *InPEC*; and amikacin, 4.17% in *ExPEC* and 0% in *InPEC*). This result is in disagreement with other authors who have reported higher levels of resistance [39]. Notably, the MDR rate is 80.7% of the isolates from healthy waterfowls in Esatern China [40], whereas the MDR rate is 29.31%, and XDR rate is 10.34% in our study. MDR patterns were more diverse in *ExPEC* isolates compared with *InPEC* isolates. Pan et al. [41] characterized a multidrug-resistant region in an F33: A-: B-plasmid carrying bla*TEM-1*, bla*CTX-M-65*, *rmtB*, and *fosA3* in an isolate from an avian *E. coli* strain of ST117. A draft genome sequence of a *CTX-M-8*, *CTX-M-55*, and *FosA3* co-producing *E. coli* ST117-B2 was isolated from a human symptomatic carrier [33].

Then, we analyzed the relationships between antimicrobial resistance phenotypes and resistance genes in our *ExPEC* and *InPEC* isolates. As previouly reported, quinolone resistance was due to mutations in *gyrA* in 97% isolates [42]. Resistance phenotypes of ciprofloxacin and levofloxacin were associated to S83L + D87N mutations among all Enterobacteriaceae (*P* < 0.001, [43]). In this study, 62.06% (36/58) of the strains have the gyrA S83L mutation, and 29.31% (17/58) of the strains have the gyrA D87N mutation, resistance phenotypes of ciprofloxacin among 27.59%(16/58) isolates were all harbored mutation S83L + D87N. Nevertheless, gyrA(S83L) single mutation may induce resistance to enorfloxacin and nalidixic acid. Therefore, we need to detected the mutation of gyrA in clinical *E. coli* isolates, with caution to reduce the development of quinolones resistance. Streptomycin resistance was attributable to the *aadA*, *strA*, and *strB* genes [44]. And in this study, we have found that *strA* gene was in 82.76% of *E. coli* isolates (48/58). Among 48 phenotypically identified streptomycin isolates, the prevalence *strA* and *strB* genes was 100%(48/48) and 64.58%(31/48), respectively. And this results was consistant with van Overbeek’sstudy [45]. ESBL enzymes confer resistance to penicillins, cephalosporins, monobactams and other antibiotic classes. The *TEM*, *CTX-M* and *SHV* types have been recognized as the most prevalent ESBL genes conferring antibiotic resistance in pathogenic bacteria worldwide [46–48]. Kpoda DS [49] revealed the most prevalent ESBL resistance genes were *CTX-M* (40.1%), *TEM* (26.2%) and *SHV* (5.9%) in *Enterobacteriaceae*. In this study, among 33 phenotypically identified β-lactamases isolates, *CTX-M* was the most prevalent gene (56.90%), followed by *SHV* at 41.38%, *OXA* at 5.17% and *TEM* at 1.72%. according to the results, we can illustrate the aminoglycosides resistance phenotypes harbored the strA and strB genes, gyrA(S83L) single mutation may induce resistance to enorfloxacin and nalidixic acid. Ciprofloxacin resistance may lead *gyrA* (S83L + D87N) double mutation in *E. coli* isolates.

## Conclusions

In this study, we find that the extra-intestinal *E. coli* mainly belong to the phylogenic groups (B2/D), which was considered as *ExPEC*. The dominant STs of *ExPEC* included ST117, ST297, ST93, ST1426, and ST10, while the dominant ST of *InPEC* was ST117. In addition, the multi-resistance rate was 51.72% in all of *ExPEC* and *InPEC* isolates. Aminoglycosides resistance was attributable to the *strA*, and *strB* genes. Quinolonones and fluoroquinolones was attributable to the *mutation of gyrA*(S83L/D87N). The prevalence of MDR and XDR of the tested *E. coli* isolates was 29.31% (17/58) and 10.34%(6/58), respectively. Virulence genes of *iss* and *iutA* were significantly different between *InPEC* and *ExPEC*, which may be virulence traits to distinguish the virulence of *E. coli* isolates*.*

## Methods

### Isolation and identification of *E. coli*

A total of 200 *E. coli* isolates from chicken were collected from Xiangyang, Shishou, Jiangxia, Jinzhou and Tianmen in Central China from 2019 to 2020. *InPEC* was isolated from feces and intestinal tissues, and *ExPEC* was isolated from heart, blood, liver, lung, eye and brain tissues. Samples were streaked onto MacConkey and eosin-methylene blue agar (hopebio Co. Ltd., Qingdao, China) plates and incubated for 24 h at 37 °C. *E. coli* isolates were confirmed using standard biochemical and bacteriological methods [50]. The assumed *E. coli* were confirmed by PCR amplification of the *phoA* gene as described previously [51], using the following primers: *phoA*-F, 5′-GCACTCTTACCGTTACTGTTTACCCC-3′, *phoA*-R, 5′--3′- TTGCAGGAAAAAGCCTTTCTCATTTT, 1001 bp.

### Ethics statement

The sample of sick chickens from farms were euthanized by cervical dislocation and then dissected with aseptic surgical techniques. All experimental protocols were approved by the Ethics Committee of Hubei Academy of Agricultural Sciences. All methods were carried out in accordance with the regulation of Hubei Province Laboratory Animal Management Regulations-2005. Collection of organ samples from the farms complies with the ARRIVE guidelines (https://arriveguidelines.org) for the reporting of animal experiments.

### Determination of phylogenetic groups

Genomic DNA was extracted from *E. coli* isolates using the boiling method as previously described [52]. Phylogenetic groups of *E. coli* isolates were determined (groups A, B1, B2, D) using the triplex PCR by amplifying the following gene targets of *chuA*, *yjaA*, and *TspE4. C2* as described previously [53]. For each PCR reaction, 2 μL samples of cell suspension of the *E. coli* strains were prepared in 25 μL of sterile deionized water.

### Antimicrobial susceptibility testing

A total of 16 commercially available antibiotic discs (Binhe Microorganism Reagent Co. Ltd., Hangzhou, China) for veterinary and human use, including Extend-spectrum cephalosporins: Ceftazidime (CAZ, 30 μg), Cefepime (FEP, 30 μg), Cefotaxime (CEF, 75 μg); Non-extend spectrum cephalosporins: Cefuroxime (CXM, 30 μg), Cephalothin (KF, 30 μg); Penicillins: Piperacillin (TZP, 100 μg); Cephamycins: Cefoxitin (FOX, 30 μg); Monobactams: Aztreonam (AZT, 30 μg); Aminoglycosides: Streptomycin (STR, 300 μg), Gentamicin (CN, 120 μg), Spectinomycin (SH, 25 μg); Fluoroquinolones (Ciprofloxacin (CIP, 5 μg), Enrofloxacin (ENR, 5 μg); Quinolones: Nalidixic acid (NA, 30 μg), were prepared for antimicrobial susceptibility testing. The diameter of the inhibition zones of 16 commercially available antimicrobial drugs were determined as susceptible, intermediate, or resistant by the Clinical and Laboratory Standards Institute protocols [54]. *E. coli* ATCC 25922 in the test was used for quality control. The tested strains are classified into MDR, XDR and PDR as previously described by Magiorakos [17].

### MLST analysis

Gene amplification and sequencing of the internal fragments from seven specific housekeeping genes (*adk*, *fumC*, *gyrB*, *icd*, *mdh*, *purA*, and *recA*) were performed using PCR as described previously [55]. The allelic profiles of the seven gene sequences and the STs were uploaded to the EnteroBase database (http://mlst.warwick.ac.uk/mlst/dbs/Ecoli), and the *E. coli* STs were matched. In addition, we performed in silico phylogroup typing and MLST [56]. Phylogenetic and genomic diversity of *E. coli* strains was constructed by using the UPGMA cluster analysis with START Version 2 (http://pubmlst.org/software/analysis/start2/).

### Detection of virulence-associated genes

DNA was extracted from the *E. coli* isolates and reference strains using the boiling method. The *E. coli* isolates were analyzed for the presence of the target genes using PCR and sample sequencing. The primer sequences of 13 virulence genes are shown in Table 5. All PCR reactions were carried out on 25 μl samples containing 12.5 μl mix (Vazyme Biotech, Nanjing, China), 8.5 μl ddH_2_O, 1 μl each of forward and reverse primer, and 2 μl DNA template. PCR amplifications were carried out in a GeneAmp PCR System 9700 (Applied Biosystems, Darmstadt, Germany) under the following conditions: initial denaturation at 95 °C for 5 min, 30 denaturation cycles at 95 °C for 30 s, annealing at 56 °C for 45 s, amplification at 72 °C for 30 s, and a final extension at 72 °C for 10 min. PCR amplification products were separated by electrophoresis on 1% agarose gel, stained with ethidium bromide, and visualized using a GelDoc XR System (Bio-Rad, Shanghai, China).

**Table 5:** The information of primers used in this study

Gene	Sequence (5^,^-3^,^)	Size (bp)	Description	Reference/Accession
*cnf*	AAGATGGAGTTTCCTATGCAGGAG TGGAGTTTCCTATGCAGGAG	498	Cytotoxic necrotizing factor	Johnson & Stell. (2000)
*feoB*	AATTGGCGTGCATGAAGATAACTG AGCTGGCGACCTGATAGAACAATG	470	Ferrous iron transporter	Yamamoto et al. (1995)
*irp2*	AAGGATTCGCTGTTACCGGAC AACTCCTGATACAGGTGGC	413	Yersiniatbactin biosynthesis	Ewers et al. (2005)
*iroD*	AAGTCAAAGCAGGGGTTGCCCG GACGCCGACATTAAGACGCAG	665	Catecholate siderophore receptor	Johnson. (2000)
*ibeA*	AGGCAGGTGTGCGCCGCGTAC TGGTGCTCCGGCCAACCATGC	170	Invasion of brain endothelium	Johnson & Stell. (2000)
*fimH*	TGCAGAACGGATAAGCCGTGG GCAGTCACCTGCCCTCCGGTA	508	Type 1 fimbriae	Johnson & Stell. (2000)
*LT*	TATCCTCTCTATATGCACAG CTGTAGTGGAAGCTGTTATA	480	Enterotoxins	Osman et al. (2012)
*kpsM*	CATCATCAAATGGCAAGA AAGCAGTATCGGCAGGAC	394	capsular polysaccharide synthesis	AF007777.1
*ompT*	TCATCCCGGAAGCCTCCCTCACTACTAT TAGCGTTTGCTGCACTGGCTTCTGATAC	496	Outer member protein	MG149556.1
*iutA*	GGCTGGACATCATGGGAACTGG CGTCGGGAACGGGTAGAATCG	302	Iron acquisition system	JX466848.1
*iss*	CAGCAACCCGAACCACTTGATG AGCATTGCCAGAGCGGCAGAA	323	serum resistance	AF042279.1
*hlyA*	AACAAGGATAAGCACTGTTCTGGCT ACCATATAAGCGGTCATTCCCGTCA	1177	α-Hemolysin	Yamamoto et al. (1995)
*ompA*	AAATACGGTAGAGTCAGGTGG CGTTCACGCTTAATAAATGG	330	Outer membrane protein	FJ158545.1
*TEM*	CATTTCCGTGTCGCCCTTATTC CGTTCATCCATAGTTGCCTGAC	800	β-lactamases resistance	Yao F et al.(2007)
*SHV*	AGCCGCTTGAGCAAATTAAAC ATCCCGCAGATAAATCACCAC	713		
*OXA*	GGCACCAGATTCAACTTTCAAG GACCCCAAGTTTCCTGTAAGTG	564		
*CTX-M*	CGCTTTGCGATGTGCAG ACCGCGATATCGTTGGT	550		
*strA*	GCCAAAGGTCGAGGTGTGG CCAGTTCTCTTCGGCGTTAG	515	Aminoglycosides resistance	van Overbeek et al. (2002)
*strB*	GACTCCTGCAATCGTCAAGG GCAATGCGTCTAGGATCGAG	560		
*aadA*	CAGCGCAATGACATTCTTGC GTCGGCAGCGACATCCTTCG	295		
*gyrA*	CGATGTCGGTCATTGTTG CTTCCGTCAGGTTGTGC	496	Fluoroquinolones/Quinolones resistance	MF374502.1

#### Detection of resistance genes

PCR were used to identify genes responsible for resistance to beta-lactams, aminoglycosides, quinolones and fluquinolones. In total, seven antimicrobial-resistant genes, including *TEM*, *SHV*, *OXA*, *CTX-M*, *strA strB*, and *aadA* were detected as previously described [45, 57]. To detect the mutations in *gyrA* gene, *gyrA* was amplified and sequenced.

### Statistics analysis

All experiments were analyzed using unpaired, two-tailed Student’s t-test. Statistical significance is determined when *P* < 0.05. All analyses were conducted using the IBM SPSS Statistics 19 software (IBM, USA).

## Supplementary Information

**Additional file 1 Supplement Table 1**. The results of resistance phenotypes and antimicrobial resistance genes detected in 58 *E. coli* isolates.

**Additional file 2 Supplementary Fig. 1** Raw figure of the Fig. 1.

## Data Availability

The datasets generated and/or analysed during the current study are available in the GenBank repository, Accession number from MZ346046(https://www.ncbi.nlm.nih.gov/nuccore/MZ346046) to MZ346423(https://www.ncbi.nlm.nih.gov/nuccore/MZ346423). The other datasets generated and analyzed during the current study are available from the corresponding author on reasonable request.

## Abbreviations

*E. coli*: *Escherichia coli*
*InPEC*: Intestinal Pathogenic *Escherichia coli*
*ExPEC*: Extra-intestinal Pathogenic *Escherichia coli*
MLST: Multilocus Sequence Typing
STs: Sequence types
