# Supplementary figures and images for: Genetic diversity and multidrug resistance of phylogenic groups B2 and D in InPEC and ExPEC isolated from chickens in Central China
Source: BMC Microbiol. 2022 Feb 18;22:60. doi: 10.1186/s12866-022-02469-2 (PMC8855568; doi:10.1186/s12866-022-02469-2)

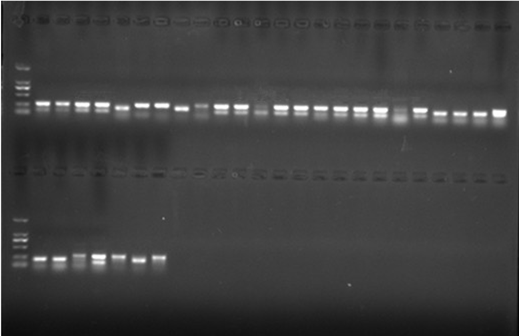


**Supplementary Fig1.** Raw figure of the figure 1.

Supplement: Supplementary file 2 — Additional file 2 Supplementary Fig. 1 Raw figure of the Fig. 1. [file 12866_2022_2469_MOESM2_ESM.docx]
